# Pain perception in women with menstrually-related migraine

**DOI:** 10.1177/0333102420966977

**Published:** 2020-10-21

**Authors:** Katie M Linstra, Khatera Ibrahimi, Daphne S van Casteren, Marieke JH Wermer, Gisela M Terwindt, Antoinette MaassenVanDenBrink

**Affiliations:** 1 Division of Pharmacology and Vascular Medicine, Department of Internal Medicine, Erasmus MC, Rotterdam, The Netherlands; 2 Department of Neurology, Leiden University Medical Center, Leiden, The Netherlands

**Keywords:** Headache, menstrual cycle, sex hormones, estrogen

## Abstract

**Background:**

Cyclic hormonal fluctuations influence migraine incidence and severity. Previously, we described reduced menstrual cyclicity in estradiol levels and dermal blood flow reaction to capsaicin in female migraineurs. It is unclear whether pain perception in women with migraine is influenced by the menstrual cycle.

**Methods:**

Women with menstrually-related migraine (n = 14), healthy age-matched controls (n = 10) and postmenopausal women (n = 15) were asked to grade trigeminal and non-trigeminal painful stimuli on a numeric pain rating scale on menstrual cycle day 19–21 (mid-luteal) and day 1–2 (early follicular).

**Results:**

In women with menstrually-related migraine, trigeminal pain remained low throughout the cycle. Controls showed increased trigeminal pain during the mid-luteal phase compared to the early follicular phase. Changes throughout the cycle were significantly different between women with MRM and controls.

**Conclusion:**

The compromised menstrual cyclicity of pain perception in women with menstrually-related migraine parallels our earlier findings on estradiol levels and dermal blood flow.

## Introduction

Migraine manifestation is closely associated with fluctuations of sex hormone levels (1). Migraine onset typically occurs around menarche and prevalence is highest among women in their fertile period, decreasing again after menopause. Migraine attack frequency and severity change during pregnancy and lactation, the use of hormonal oral contraceptives as well as the menstrual cycle, resulting in frequent perimenstrual attacks and the menstrually-related migraine (MRM) subtype (1,2). One of the most important fluctuating sex hormones affecting migraine is estradiol (1). Perimenstrual migraine attacks that occur immediately following the natural drop in estradiol levels are reported to be more painful and disabling, and are more often accompanied by allodynia (2,3).

We previously reported that menstrual cyclicity of estradiol levels and forehead dermal blood flow (DBF) response to capsaicin, a measure for trigeminal nerve-mediated microvascular reactivity, are compromised in women with MRM (4). Besides this neurovascular mechanism, pain perception may also be influenced by changes during the menstrual cycle. Therefore, with this case-control study, we aimed to explore differences in pain perception in women with MRM after peak estradiol levels drop in the menstrual cycle (day 1–2; early follicular phase) compared to an interval with relatively steady, high estradiol levels (day 19–21; mid-luteal phase). We included healthy women without migraine as controls and postmenopausal women as a reference group without menstrual cyclicity. Since the sensation of pain during migraine attacks is attributed to activation of the trigeminovascular system, we further distinguished between pain perception in the trigeminal and non-trigeminal dermatomes. The results indicate whether, apart from trigeminal nerve-mediated microvascular reactivity, pain perception is also affected by the menstrual cycle.

## Methods

### Subjects

Premenopausal women with MRM (n = 14), age-matched healthy female controls (n = 10) and postmenopausal women (n = 15) were recruited between March 2011 and August 2012. Women with MRM were recruited through the Leiden University Migraine Neuro-Analysis (LUMINA) headache database (see supplemental material) (5). MRM was diagnosed according to the International Classification of Headache Disorders (ICHD-3 beta) criteria (6). MRM patients with prophylactic antimigraine treatment were excluded, and included participants who refrained from using acutely acting antimigraine therapy 48 hours before visits. Women with known cyclical irregularities or comorbidity and women using hormonal contraceptives were excluded. Basic parameters such as length, weight and blood pressure and migraine characteristics were assessed. The recruitment and selection design is described in further detail in our previous publication (4).

### Assessment of pain

Women with MRM and healthy women were invited for visit 1 at day 19–21 after the first day of menstruation (mid-luteal phase) and for visit 2 at day 1–2 of their following menstruation (early follicular phase). Postmenopausal women were invited for two visits with a 7–10 day interval. All participants followed a study protocol including venipuncture and forehead and forearm dermal blood flow measurements. During both visits, participants were asked to assign pain scores according to the numeric pain rating scale (0–10 NPRS) when certain painful stimuli were applied. Since the forehead skin is innervated by the trigeminal nerve, stimuli that were applied there were considered as stimuli to the trigeminal nervous system. These included electrical stimulation and topical application of different concentrations (0.06 mg/mL and 6.0 mg/mL) of capsaicin, a TRPV1 receptor agonist and the active component of chili peppers. As stimuli to the non-trigeminal nervous system, pain scores were assessed at electrical stimulation to the skin in the neck, application of high pressures with a sphygmomanometer around the upper arm for 5 min and during venipuncture (4).

### Statistical analyses

For each subject, trigeminal and non-trigeminal pain scores were calculated as the average of all measurements per visit. Differences within groups were analysed using Wilcoxon matched pair signed rank test and differences between groups per visit and across the menstrual cycle (Δ) were analysed using the Mann Whitney and Kruskal-Wallis tests, separately, for trigeminal and non-trigeminal pain scores. Statistical analyses were performed using SPSS 25.0 and GraphPad Prism 8. *p* values < 0.05 were considered to indicate significant differences.

## Results

Body mass index, blood pressure and heart rate were not statistically different between groups and were within the normal range (Table 1). Mean age was highest in postmenopausal women (60 ± 5 years), and there was no statistically significant difference between women with MRM (33 ± 7 years) and controls (38 ± 7 years) (*p* = 0.446). Most women with MRM suffered from 13–54 migraine attacks per year (1–4.5 attacks per month), with an average disease duration of 15 years. Seven women with MRM were in the ictal phase during either visit 1 or visit 2, of which four women were ictal during both visits.

**Table 1.** Clinical and headache characteristics^a^.

Variable	Migraine patients(n = 14)	Controls(n = 10)	Postmenopausal women(n = 15)
Age, years	33 ± 7 (21–44)	38 ± 7 (26–45)	60 ± 5 (50–68)
BMI, kg/m^2^	22.7 ± 2.8	23.7 ± 1.5	23.5 ± 2.4
BP, mmHg			
Systolic	110 ± 8	109 ± 8	117 ± 9
Diastolic	67 ± 7	65 ± 6	70 ± 7
HR, bpm	62 ± 9	65 ± 8	62 ± 9
Age at migraine onset, y	19 ± 7 (8–36)	–	–
Disease duration, y	15 ± 7 (6–31)	–	–
Attack frequency^b^			
1–2	0	–	–
3–6	2	–	–
7–12	3	–	–
13–54	9	–	–
Ictal during visit			
Visit 1	7 (50)	–	–
Visit 2	7 (50)	–	–
Both	4 (29)	–	–
Pain scores^c^			
Trigeminal			
Visit 1	1.9 ± 0.3	2.9 ± 0.4	2.0 ± 0.3
Visit 2	2.0 ± 0.3	1.9 ± 0.4	1.7 ± 0.2
Non-trigeminal			
Visit 1	2.8 ± 0.4	3.1 ± 0.5	2.7 ± 0.3
Visit 2	2.8 ± 0.4	3.4 ± 0.4	2.4 ± 0.3

BMI: body mass index; BP: blood pressure; HR: heart rate; bpm: beats per minute.

^a^Data are represented as mean ± SD or number of subjects (%).

^b^Number of attacks per year.

^c^Mean ± SEM.

**Figure 1.**
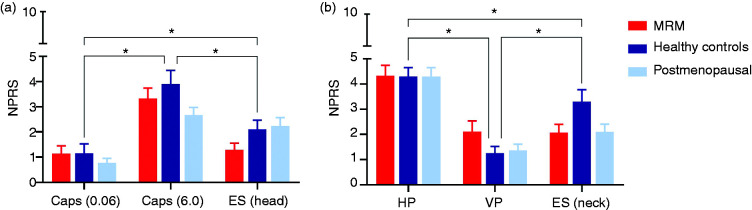
Comparison of pain stimuli. Pain scores in numeric pain rating scale (0–10 NPRS) for trigeminal ((a), left panel) and non-trigeminal ((b), right panel) pain stimuli: Capsaicin 0.06 mg/ml, capsaicin 6.0 mg/ml, electrical stimulation to the forehead (ES head), high pressures applied with sphygmomanometer (HP), venipuncture (VP) and electrical stimulation to the neck (ES neck). Controls (dark blue) and patients with MRM (red) and postmenopausal women (light blue). *Significant difference in NPRS between painful stimuli (*p* < 0.05).

**Figure 2.**
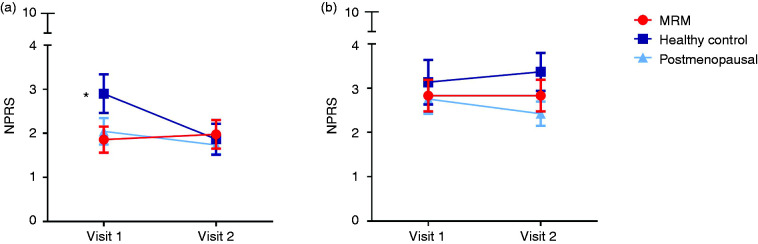
Comparison of pain between groups and visits. Pain scores in numeric pain rating scale (0–10 NPRS) for trigeminal ((a), left panel) and non-trigeminal ((b), right panel). For controls (dark blue ■) and patients with MRM (red ●): Visit 1 = days 19–21 of the menstrual cycle and visit 2 = days 1–2 of menstruation. For postmenopausal women (light blue ▲): Visit 1 and visit 2 planned randomly with 7–10 days in between. *Significant difference in NPRS between visit 1 and visit 2 for healthy controls (*p* = 0.003).

Comparing the different pain stimuli, capsaicin (6.0 mg/ml) and high pressures with a sphygmomanometer induced the highest pain grades, followed by electrical stimulation, venipuncture and capsaicin (0.06 mg/ml) (Figure 1). When discriminating between study visits to assess changes during the menstrual cycle, healthy women scored trigeminal pain significantly higher on visit 1 (day 19–21, mid-luteal phase) compared to visit 2 (day 1–2, start menstruation) (*p* = 0.003) (Figure 2, Table 1). The increase in trigeminal pain score of healthy controls between visits (Δ: 1.0 ± 0.3, (mean ± SEM)) was significantly different from the stable responses in MRM patients (Δ: −0.1 ± 0.3) (*p* = 0.011), but did not differ significantly from postmenopausal women or for non-trigeminal pain. Trigeminal pain scores were equally low on both visits for women with MRM. This was also found in post-menopausal women. No significant differences in pain perception were observed, either between visits or groups, for the non-trigeminal pain stimuli (Figure 2).

## Discussion

In this study, we assessed trigeminal and non-trigeminal pain perception in women with MRM, healthy controls, as well as postmenopausal women. Women with MRM reported equally low pain grades throughout the menstrual cycle, in both the trigeminal and non-trigeminal dermatome. Results were similar for post-menopausal women. In healthy controls, reported pain for the trigeminal dermatome, but not for non-trigeminal pain, showed a significant decrease from the mid-luteal phase to the start of menstruation, which was significantly different from the stable responses in women with MRM over these phases.

Literature on pain perception throughout the menstrual cycle is conflicting. A small study using laser-evoked pain sensations reported cyclical changes, but differently from our study, this was found in both trigeminal and non-trigeminal dermatomes and in women with and without migraine (7). A direct comparison with our study is hampered by the fact that different phases of the menstrual cycle were investigated. In line with our findings on non-trigeminal pain, another study reported that non-trigeminal pain thresholds upon electrical stimulation did not differ between migraineurs and non-migraineurs and did not vary throughout the menstrual cycle (8). However, a recent review reported no association of the menstrual cycle phase to either trigeminal or non-trigeminal pain perception in healthy women, although imaging studies showed menstrual cycle-sensitive activation of brain regions associated with pain (9).

Though previous data may seem conflicting, our observation of a compromised menstrual cyclicity in pain perception in women with MRM does substantiate our earlier findings of reduced cyclicity in estradiol levels and forehead DBF responses to capsaicin (4). Both pain perception and estradiol levels in women with MRM are relatively low during the mid-luteal phase compared to those of healthy women, and steadily remain so throughout the cycle. Our findings may indicate a decreased sensitivity of one or more factors in the trigeminovascular system to cyclic changes in women with MRM. The mechanisms behind the influence of the menstrual cycle on the trigeminal system remain uncharted, but it is likely that sex hormones, specifically estradiol, play a major role (10,11). Decreased pain thresholds are generally associated with a withdrawal of estradiol, not with an absolute high level of the sex hormone in itself (1,9). Thus, a reduction in fluctuating estradiol levels in MRM patients as compared to women without migraine (4) could be responsible for the reduced cyclicity in trigeminal pain sensation. Trigemino-specific menstrual cycle dependence may be explained by estrogenic modulation through a myriad of candidate receptors and pathways, including serotonergic and GABA-ergic pathways, and trigeminovascular CGRP, TRPV1 and P2X3 receptors (10,11). The exact physiological mechanisms remain elusive, as estradiol has a complex genomic and non-genomic influence on both pro- and anti-nociception.

A noteworthy limitation to our study is the potential bias in women with MRM, whose experience with severe migraine pain may have compromised their assessment of the painful stimuli. Moreover, decreased pain thresholds and allodynia have been reported during the ictal phase (12), and some of the MRM patients were ictal during the study visits (Table 1). However, since our results show no increased pain scores in women with MRM in either phase compared to controls, and the number of ictal patients was identical during both study visits, it is unlikely that this has affected our study. Additionally, pain was not measured using specifically designated methods, possibly resulting in relatively low overall scores and an indirect comparison between trigeminal and non-trigeminal pain. Besides, it could be argued that monitoring more than one menstrual cycle using more frequent intervals and urinary LH measurements to determine phase would improve the ability to assess the cyclicity of our endpoints. Finally, our sample size was limited, which means our results should be interpreted accordingly. Notwithstanding the shortcomings of our study, our results seem to indicate that trigeminal, but not non-trigeminal pain perception fluctuates throughout the menstrual cycle, and is affected in women with MRM. This may provide additional evidence for an altered cyclic sensitivity of the trigeminovascular system in these women. Whether our findings are part of the pathophysiological basis of MRM, and could lead to altered therapy responses in these women, remains to be seen.

## Clinical implications

- Women with menstrually related migraine appear to have a compromised menstrual cyclicity in trigeminal pain perception.
- This is in accordance with earlier findings describing a compromised menstrual cyclicity in estradiol levels and forehead dermal blood flow response to capsaicin in women with MRM compared to controls.
- Our finding of an altered trigeminal pain sensitivity in women with MRM may aid in our understanding of MRM pathophysiology and could be of influence on response to therapy in these women.

## Supplemental Material

sj-pdf-1-cep-10.1177_0333102420966977 - Supplemental material for Pain perception in women with menstrually-related migraine Click here for additional data file. Supplemental material, sj-pdf-1-cep-10.1177_0333102420966977 for Pain perception in women with menstrually-related migraine by Katie M Linstra, Khatera Ibrahimi, Daphne S van Casteren, Marieke JH Wermer, Gisela M Terwindt and Antoinette MaassenVanDenBrink in Cephalalgia
